# Study on Attenuation Characteristics of Acoustic Emission Signals with Different Frequencies in Wood

**DOI:** 10.3390/s22165991

**Published:** 2022-08-11

**Authors:** Feilong Mao, Saiyin Fang, Ming Li, Changlin Huang, Tingting Deng, Yue Zhao, Gezhou Qin

**Affiliations:** 1 School of Machinery and Transportation, Southwest Forestry University, Kunming 650224, China; 2 Key Laboratory of Advanced Perception and Intelligent Control of High-End Equipment of Ministry of Education, Anhui Polytechnic University, Wuhu 241000, China; 3 School of Electrical Engineering, Anhui Polytechnic University, Wuhu 241000, China

**Keywords:** wood, NDT, acoustic emission, frequency, amplitude attenuation, energy attenuation model

## Abstract

To study the effect of frequency on the attenuation characteristics of acoustic emission signals in wood, in this paper, two types of hard wood and soft wood were studied separately, and the energy attenuation model of the propagation process of AE sources with different frequencies was established. First, using the piezoelectric inverse effect of the AE sensor, an arbitrary waveform generator was used to generate frequency-tunable pulses in the range of 1 kHz to 150 kHz as the AE source, where the AE source energy could be regulated by the output voltage level. Then, five AE sensors were placed at equal intervals of 100 mm on the surface of the specimen to collect AE signals, and the sampling frequency was set to 500 kHz. Finally, the energy value of AE signal of each sensor was calculated based on the AC principle, and the energy attenuation model was established by exponential fitting. The results showed that both the amplitude and energy of the AE signals of different frequencies showed negative exponential decay with the increase of propagation distance, and, at the same frequency, the change of AE source energy level had no significant effect on its attenuation rate. Compared with hard wood, the energy attenuation of the AE signal of soft wood was more sensitive to the change of frequency, and, at the same frequency, the attenuation rate of soft wood was smaller than that of hard wood.

## 1. Introduction

When a material is subjected to an external or internal force, the local source transitions to a low-energy steady-state by quickly releasing the energy. This phenomenon of releasing strain energy by generating transient elastic waves is called acoustic emission [1] (AE), which is, essentially, an elastic wave with different modes and frequencies [2,3]. AE technology is widely used in the field of engineering safety inspection [4] as an important high-sensitivity dynamic nondestructive testing(NDT) method that can monitor materials in real-time and evaluate their fatigue cracking [5], energy attenuation [6], and other characteristics without damaging them.

As an environmentally friendly and renewable material widely used in people’s daily life, wood is often used as a construction material, and it is particularly important to dynamically inspect and evaluate its health condition without damaging it, so AE Technology has also been widely used in the field of wood science [7,8,9] in recent years. El-Hadad et al. [10] used AE Technology to study the propagation characteristics of AE signals in wood and showed that their propagation characteristics were affected by several factors. Many scholars have conducted numerous studies on this subject, analyzing the effects of wood density [11], moisture content [12], and cracks [13] on the AE signal propagation characteristics. Meanwhile, the AE signal propagation law in anisotropic materials, such as most bamboo [14] and glass fiber [15], was also studied, and it was shown that the AE characteristic parameters can effectively characterize the damage state, as well as the crack changes in the materials.

In recent years, some results have been achieved in the study of AE signal attenuation in other construction materials. For example, Zhao et al. [16] analyzed the variation law of the characteristic parameters during the decay of AE signals in medium-grained sandstones by spectral conversion, mathematical statistics, and curve fitting. Wu et al. [17] analyzed the effect of aggregate particle size in concrete on AE signal propagation and attenuation, using AE technology. The attenuation phenomenon of the AE signal was also used to evaluate the state of the composite material. Maillet et al. [18] assessed damage and life prediction of composites using energy attenuation of AE signals. Morscher et al. [19] used the attenuation of the AE waveform to characterize the size of the crack opening in a composite matrix. Techniques based on AE signal characterization, spectrum analysis, and pattern recognition are widely used for feature extraction and analysis of attenuated signals [20,21,22]. Li et al. [23] studied the propagation velocity of surface transverse and internal longitudinal waves and their energy attenuation in wood by PLB (pencil-lead break) tests, and showed a significant exponential attenuation of AE energy in the wood.

At present, studies on the propagation characteristics of AE signals in wood mainly use PLB as the AE source, while the frequency domain components of AE signals generated by PLB are complex, and the attenuation of different frequency domain components of AE signals cannot be clarified. For this reason, this paper used single-channel arbitrary waveform generator to generate frequency adjustable pulse string signal to simulate AE source, and its pulse frequency varied from 1 kHz to 150 kHz. The attenuation characteristics of AE signals at different frequencies were studied by using hard wood *Ulmus pumila*, *Zelkova schneideriana* and soft wood *Cunninghamia lanceolata*, *Pinus sylvestris var. mongolica* solid-sawn timber as test materials and five sensors arranged equidistantly on the surface of the specimens. When calculating the AE signal energy, the AE signal was considered an AC signal, and the AE signal energy was defined as the thermal energy consumed by the AE signal through the unit resistance for a definite time. By comparing the amplitude and energy of AE signals collected by each sensor under different frequency AE sources and establishing their attenuation models, we provided a reference for subsequent localization studies based on the energy attenuation of AE signals at different frequencies. Therefore, the study in this paper addresses three areas:

- Introduction of test materials and experimental methods (Section 2).
- Modeling AE signal amplitude and energy attenuation (Section 3).
- Discussion of research results and outline of future research directions (Section 4).

## 2. Materials and Methods

### 2.1. Experimental Materials

This paper selected air-dried hard wood *Ulmus pumila*, *Zelkova schneideriana*, and soft wood *Cunninghamia lanceolata*, *Pinus sylvestris var. mongolica* solid-sawn timber as test materials, and manufactured four sawn timber test pieces of the same dimensions (800 × 60 × 30 mm). The grain was straight, with no surface defects. Before the experiment, the wood density was measured by DA-900CE (Shenzhen DahoMeter Instrument Co., Ltd., Shenzhen, China), according to ASTM D2395-17 [24], and the moisture content was measured by 101-0EBS drying oven (Tianjin Hongnuo Instrument Co., Ltd., Tianjin, China), according to ASTM D4442-20 [25]. For the convenience of description, the test pieces were defined as T1~T4, and the relevant parameters of each test piece are shown in Table 1.

**Figure 1:**
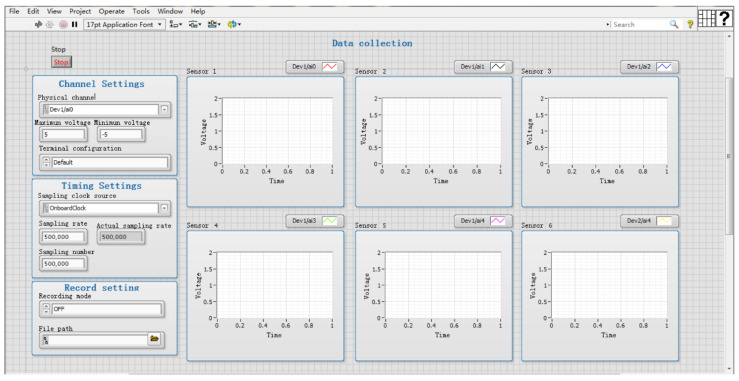
Test parameters.

A 5-channel AE signal acquisition system was built, based on LabVIEW 2017 software (National Instruments) and NI USB-6366 high-speed acquisition card (National Instruments). The sensor was an RS-2A (Beijing Soundwel Technology Co., Ltd., Beijing, China) single-ended resonant AE sensor with a signal bandwidth of 50 to 400 kHz, and the system was equipped with a PA I gain amplifier (Beijing Soundwel Technology Co., Ltd., Beijing, China) with a gain of 40 dB, which could effectively amplify the acquired AE signal. The voltage range at the output of the acquisition card used was set to −5 V, 5 V, and its sampling frequency was a maximum of 2 MHz. The current study [26,27] showed that the highest frequency of AE signal propagation in wood was around 200 kHz. According to Nyquist’s sampling theorem [28], to make the recovered analog signal undistorted, the sampling frequency should be greater than, or equal to, two times the maximum signal frequency, so the sampling frequency of each channel was set to 500 kHz during the acquisition process, as shown in Figure 1. During the test, to ensure sufficient coupling between the test piece and the sensors, to reduce the influence of the air medium during signal acquisition, and to improve the transduction efficiency of the sensor, high-temperature insulating grease was used to fill between the test piece and the sensors.

To obtain AE sources of different frequencies, the SIGLENT SDG805 (Siglent Technologies Co., Ltd., Shenzhen, China) single-channel arbitrary waveform generator was used to generate pulse string signals of different frequencies to simulate real damage to wood. Its sampling rate was 125 MSa/s, the output pulse frequency range was 500 μHz~5 MHz, and the output voltage range was 4 mV~20 V. The instruments used for the tests are shown in Table 2.

### 2.2. Experimental Methods

**Figure 2:**
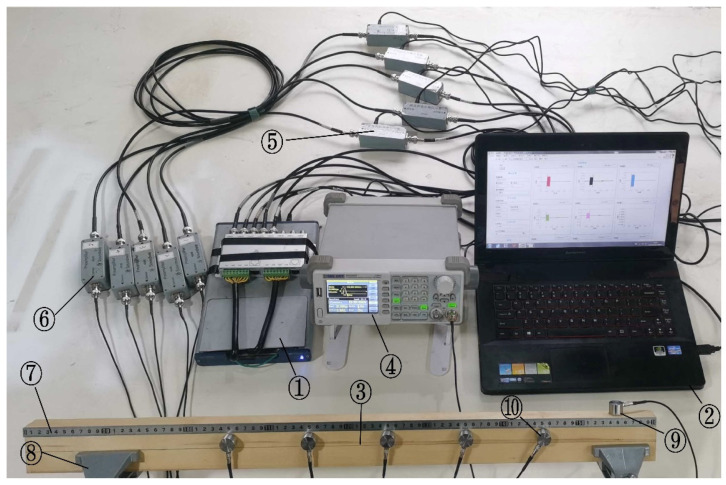
Test diagram. ① NI acquisition card ② Computer ③ Test piece ④ Arbitrary waveform generator ⑤ Signal separator ⑥ Gain amplifier ⑦ Scale ⑧ Table clamp ⑨ AE source ⑩ Sensor.

To study the attenuation characteristics of AE signals at different frequencies in wood, the experimental design scheme is shown in Figure 2. The five sensors were arranged at equal intervals from right to left, labeled as S1~S5, respectively. Where sensors S5 and S1 were respectively 250 mm and 150 mm away from the left and right end surfaces, and the remaining sensors were 100 mm apart from each other, and all sensors were co-lined. This experiment made use of the inverse piezoelectric effect of the AE sensor and used a single-channel arbitrary waveform generator to generate pulse string signals of different frequencies as the simulated AE source with frequency variations ranging from 1 kHz to 150 kHz, setting its individual pulse width to 1 μs and pulse cycle period to 1 s. The AE source was placed on the upper surface of the specimen at a distance of 50 mm from the right end face, and the AE signal with different initial energies was generated by varying its voltage level reduced from 20 V to 5 V with 5 V intervals. At the same time, the number of pulse cycles was varied to ensure that the width of the AE signal collected at different frequencies was the same. For example, at a pulse frequency of 150 kHz, the number of cycles was set to 15000, while at a pulse frequency of 100 kHz, the number of cycles was set to 10,000 When the AE signals, based on different frequencies, were collected, their amplitude and energy values were calculated. Finally, the attenuation models of AE signals with different frequencies were constructed by exponential fitting.

In the analysis of the AE signal energy, according to the thermal effect of electric current [29,30], the AE signal was considered as an AC signal and the heat generated through a unit resistance in a fixed time was taken as the AE signal energy. Its calculation formula is as follows:

(1) $$W=\int_{0}^{t}\frac{u^{2}}{R}d\tau$$

where *u* is voltage and *R* is unit resistance. Since the AE signals collected by the system were discontinuous, Equation (1) is discretized, and the interval between two data is 1/*f_s_* second. Assuming that the Zero-order retainer is used in the discretization process, i.e., the signal amplitude remains unchanged during this period, then the AE signal energy can be calculated as Equation (2).

(2) $$W=\overset{n}{\underset{i=1}{\sum }}\Delta t_{i}\cdot u_{i}^{2}=T\overset{n}{\underset{i=1}{\sum }}u_{i}^{2}$$

where *W* is the AE signal energy, *n* is the length of the data, $\Delta t_{i}=T=\frac{1}{f_{s}}$ (*i* = 1, 2..., *n*), and *f_s_* is the sampling frequency.

## 3. Results and Discussion

### 3.1. AE Signal Amplitude Attenuation Model

Using the standard pulse signal generated from an arbitrary waveform generator as an AE source, the amplitude attenuation characteristics of the AE signal at different frequencies were explored, and the attenuation model was constructed. Since wood and rock are both incomplete elastomers, wood can be referred to the rock amplitude attenuation model. According to the elastic wave theory and the quality factor Q [31], the variation of AE signal amplitude with propagation distance during propagation could be expressed as follows:

(3) $$A\left(D\right)=A_{0}e^{-\frac{\pi f}{vQ}D}=A_{0}e^{-\gamma D}$$

where *A*(*D*) is the amplitude and *A*(*D*) changes with the actual distance *D*. *A*_0_ is the initial amplitude of the AE signal, f is the input burst frequency, *v* is the propagation speed of the AE signal, *Q* is the material quality factor, and *γ* is the attenuation coefficient of the amplitude.

**Figure 3:**
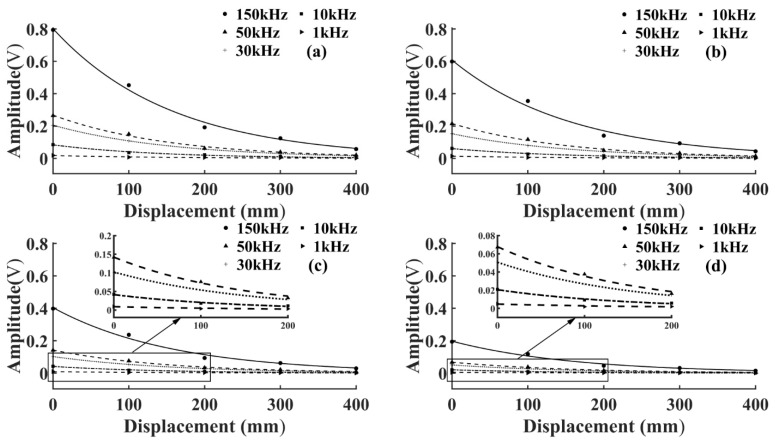
Fitting curve of AE signal amplitude attenuation of T1 specimen. (**a**) Voltage level 20 V; (**b**) Voltage level 15 V; (**c**) Voltage level 10 V; (**d**) Voltage level 5 V.

**Figure 4:**
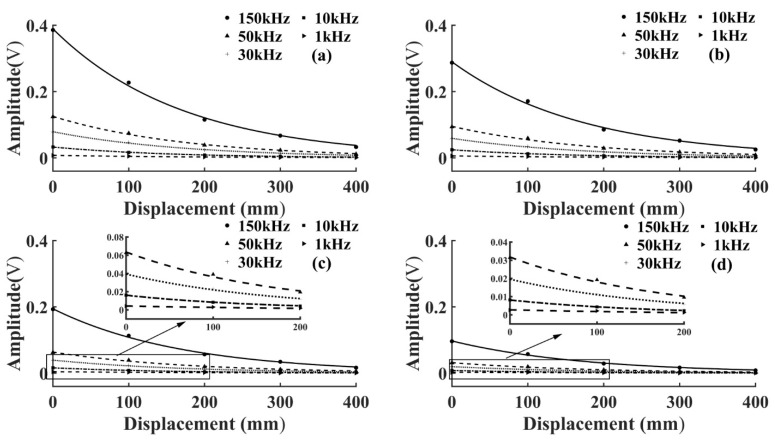
Fitting curve of AE signal amplitude attenuation of T3 specimen. (**a**) Voltage level 20 V; (**b**) Voltage level 15 V; (**c**) Voltage level 10 V; (**d**) Voltage level 5 V.

To study the amplitude attenuation law of the AE signals under different initial energy, the output pulse voltage level was changed without changing the position of the AE source. It was set from a maximum voltage amplitude of 20 V to 5 V in 5 V intervals. The amplitude of the AE signal collected by each sensor was calculated and fitted using the MATLAB fitting toolbox. To more intuitively reflect the amplitude attenuation law of AE signals at different frequencies, the amplitude attenuation curve of the same voltage level was drawn in the same graph. The goodness of fit of the amplitude fitting curves of T1 and T3 specimens was higher than 95%, as shown in Figure 3 and Figure 4.

**Figure 5:**
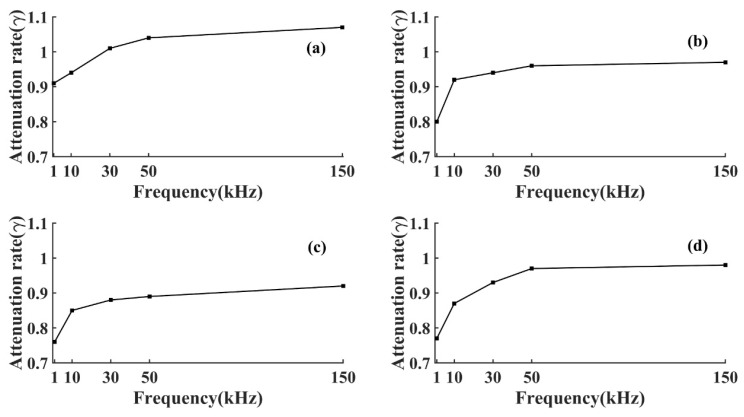
Amplitude attenuation coefficient *γ.* (**a**) T1 specimen; (**b**) T2 specimen; (**c**) T3 specimen; (**d**) T4 specimen.

As shown in Figure 3 and Figure 4, the AE signal amplitude in the same specimen increased with the increase of the pulse string voltage level at the same frequency, but the change of wood types and voltage levels did not affect the attenuation laws. At the same voltage level, the amplitude of the AE signal of the same specimen increased with the increase of frequency, which was because the higher the frequency the greater the energy carried, and the amplitude was the embodiment of energy. From Equation (3), the amplitude of the AE signal decreased exponentially with the increase of *D*, and the attenuation rate depended on the attenuation coefficient *γ*. To further study the amplitude attenuation of AE signals at different frequencies of each specimen, the effect of frequency on the amplitude attenuation rate of AE signals was analyzed in depth. The amplitude attenuation rate at different frequencies was calculated and plotted in the same graph by linear fitting, as shown in Figure 5. According to Figure 5, the wood type did not affect the variation pattern of the AE signal amplitude attenuation rate, the amplitude attenuation rate of the AE signal of each specimen increased with the increase of frequency, and the increase of the AE signal amplitude attenuation rate slowed down with the increase of frequency. This was because wood is a special viscoelastic composite biomass material, and when the stress wave propagates in a viscoelastic medium, the high frequency signal decays rapidly in a short distance, due to the viscosity of the material [32].

### 3.2. AE Signal Energy Attenuation Model

To study the energy attenuation of AE signals with different frequencies during propagation, using the piezoelectric inverse effect of AE sensor to collect the pulse signal with adjustable frequency generated by arbitrary waveform generator as AE source, the influence of signal frequency on energy attenuation law was studied, and its energy attenuation model could be constructed. Since the experimentally acquired AE signal was amplified by a 40 dB amplifier, it needed to be restored to the real AE signal, and the AE signal energy value of each sensor could be calculated by Equation (2), having unit μJ.

**Figure 6:**
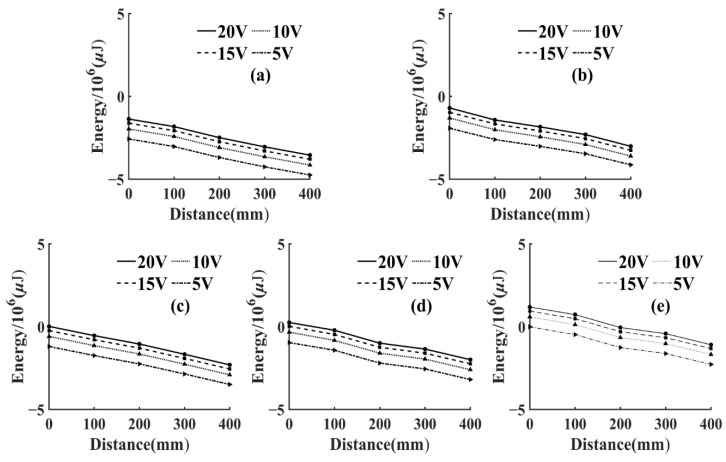
Variation of AE signal energy with propagation distance for T1 specimen. (**a**) Pulse frequency 1 kHz; (**b**) Pulse frequency 10 kHz; (**c**) Pulse frequency 30 kHz; (**d**) Pulse frequency 50 kHz; (**e**) Pulse frequency 150 kHz.

**Figure 7:**
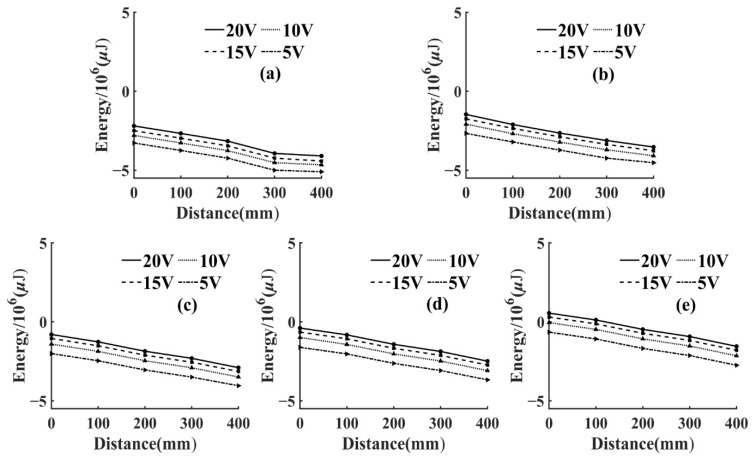
Variation of AE signal energy with propagation distance for T3 specimen. (**a**) Pulse frequency 1 kHz; (**b**) Pulse frequency 10 kHz; (**c**) Pulse frequency 30 kHz; (**d**) Pulse frequency 50 kHz; (**e**) Pulse frequency 150 kHz.

To reflect the attenuation of AE signal energy more intuitively, the energy attenuation of different voltage levels was fitted in the same figure, as shown in Figure 6 and Figure 7. Since the variation range of AE signal energy was too large, the real energy was taken from the logarithmic value as the vertical coordinate, and, thus, it could be seen that the energy attenuation law corresponding to different voltage levels of the same frequency was the same. At the same voltage level, the AE signal energy and amplitude collected by the sensor in the same specimen had a similar change law, which increased with the increase of frequency and the energy attenuated significantly with the increase of propagation distance. This was due to the fact that wood is a porous material with a large number of internal cavity structures, having good sound absorption properties [33].

**Figure 8:**
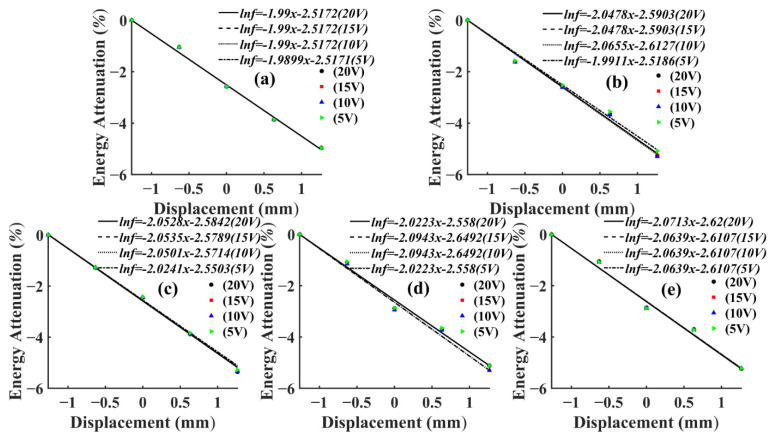
Fitting curve of AE signal energy attenuation of T1 specimen. (**a**) Pulse frequency 1 kHz; (**b**) Pulse frequency 10 kHz; (**c**) Pulse frequency 30 kHz; (**d**) Pulse frequency 50 kHz; (**e**) Pulse frequency 150 kHz.

**Figure 9:**
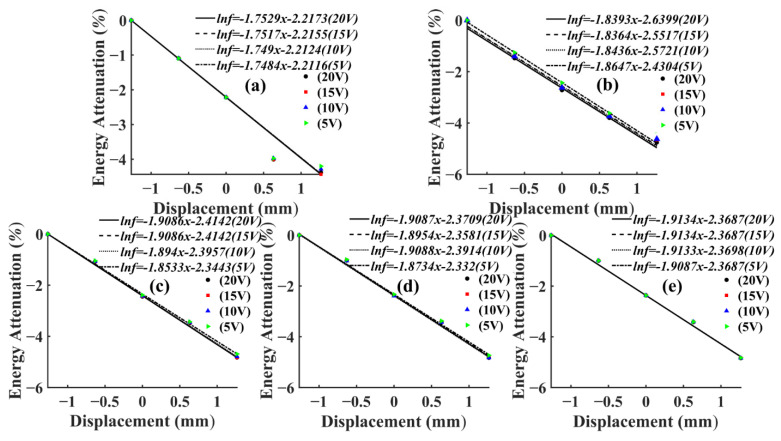
Fitting curve of AE signal energy attenuation of T1 specimen. (**a**) Pulse frequency 1 kHz; (**b**) Pulse frequency 10 kHz; (**c**) Pulse frequency 30 kHz; (**d**) Pulse frequency 50 kHz; (**e**) Pulse frequency 150 kHz.

The AE signal energy value measured by sensor S1, which was closest to the AE source, was considered as 1, and the AE signal energy values measured by the remaining sensors were normalized. From Equation (2), it can be seen that the energy of AE signal was related to the length and amplitude of the data. In this test, the length of AE signal data collected by each sensor at different frequencies was kept consistent by adjusting the number of pulse cycles, so the energy of AE signal in this paper was only related to the amplitude. Then, the AE signal energy of this paper was only related to amplitude. From Equation (3), it can be seen that the amplitude of the AE signal showed negative exponential attenuation with the increase of the propagation distance. Therefore, the energy decay process of the AE signal was fitted exponentially in this paper. Then, the AE signal energy fitting curves of T1 and T3 specimens could be obtained, as shown in Figure 8 and Figure 9.

It can be seen from Figure 8 and Figure 9 that, at the same frequency, the energy attenuation of AE signals with different voltage levels showed slight differences at local positions, but the overall attenuation law was extremely similar. This situation also appeared in the energy attenuation of T2 and T4 specimens, which indicated that changing the energy magnitude of the AE source at the same frequency had no significant effect on the overall attenuation law of the AE signal. In addition, according to Figure 8 and Figure 9, it can be seen that the attenuation rate of AE signal in *Ulmus pumila* was greater than that in *Cunninghamia lanceolata* when the frequency of the AE source was the same.

When calculating the attenuation law of AE signal energy with propagation distance, the direct fitting was prone to large fitting errors because of the significant difference between the value of distance and the value of energy after utilizing the logarithm, so a linear process of centering the distance was required. The calculation was as follows:

(4) $$x=\frac{D-mean\left(D\right)}{std\left(D\right)}$$

where *D* is the actual distance, and its value range is from 0 to 400 mm; *x* is the transformed equivalent distance, i.e., the value of the horizontal axis coordinates in Figure 8 and Figure 9; *mean*(*D*) is the expectation of *D*; *std*(*D*) is the variance of *D*. In this paper, the expectation and variance are 200 mm and 158.11 mm respectively. Therefore, the attenuation law of AE signal with equivalent propagation distance in this article could be expressed as:

(5) $$E_{i}=e^{-Kx+b}$$

where *K* is the average value of the slope of the fitted curve at different voltage levels, indicating the energy attenuation rate of AE signals at different frequencies, the larger *K* is, the faster the energy attenuation of AE signals, and the *K* values of AE signals at different frequencies of T1 and T3 specimens are shown in Table 3 and Table 4. To study the energy attenuation of AE signals at different frequencies with the actual distance *D*, substituting Equation (4) into Equation (5), then the AE signal attenuation with the actual distance *D* could be expressed as:

(6) $$E_{i}=\alpha e^{-\beta D}$$

Then, the relevant parameters of the AE signal energy attenuation fitting functions for different frequencies of T1 and T3 specimens could be obtained as shown in Table 3 and Table 4.

**Figure 10:**
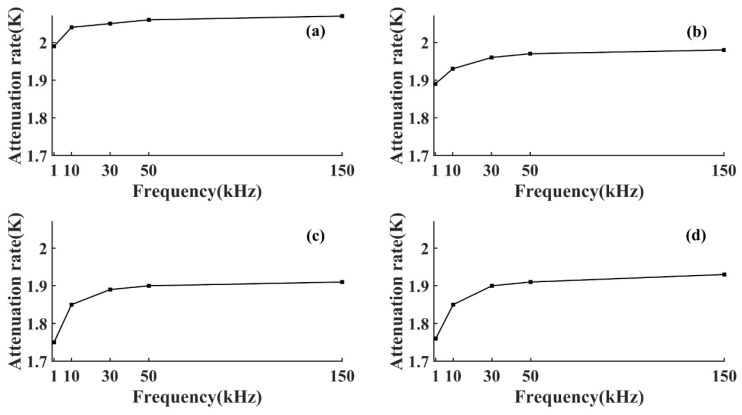
Attenuation coefficient *K.* (**a**) T1 specimen; (**b**) T2 specimen; (**c**) T3 specimen; (**d**) T4 specimen.

From Table 3 and Table 4, it can be seen that the actual attenuation rates *β* corresponding to AE signals of different frequencies were small, and the actual attenuation rates of some frequencies were relatively close to each other, which indicated that the actual attenuation rates of energy at different frequencies varied less, and it was difficult to further analyze the effect of frequency on them. Therefore, to study the effect of frequency on the attenuation of AE signal energy, after converting its actual distance into equivalent distance, the actual attenuation of AE signal energy was characterized by the change of attenuation rate *K*. To further analyze the effect of frequency on the energy attenuation law of AE signals in different wood specimens, the energy attenuation rate *K* of AE signals at each frequency in different specimens was obtained by calculation and plotted in the same figure as shown in Figure 10.

It can be seen from Figure 10 that the change law of the energy attenuation rate of the AE signal was similar to that of the amplitude attenuation rate, both of which increased with frequency, similar to the results obtained from the Bucur and Böhnke study [34]. To more clearly characterize the change in *K*, the attenuation rate at the AE signal frequency of 1 kHz was used as the basis for calculating the frequency *K* increase rate *η*$(\eta =\frac{k_{i}-k_{1}}{k_{1}}\;i=10,30,50,150$) for each frequency, as shown in Table 5.

**Figure 11:**
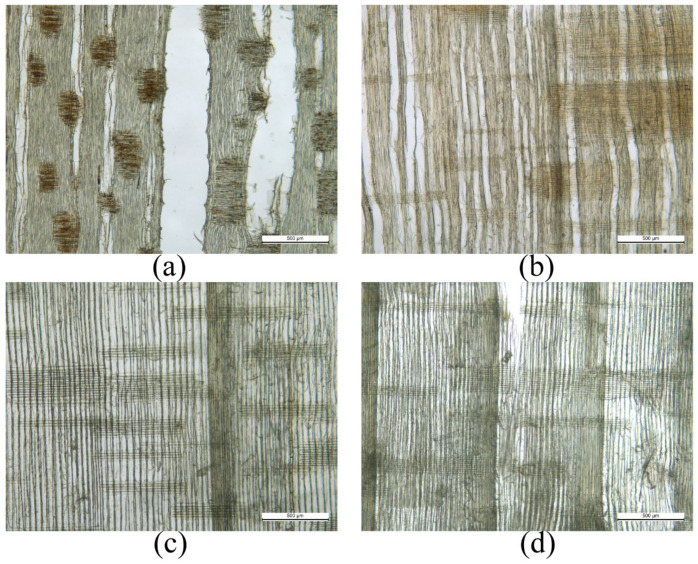
Microstructure of wood. (**a**) T1 specimen; (**b**) T2 specimen; (**c**) T3 specimen; (**d**) T4 specimen.

It can be seen from Table 5 that the increase of AE signal energy attenuation rate gradually slowed down with the increase of frequency, and the increased rate of AE signal energy attenuation rates of hard wood were all smaller than that of soft wood, which indicated that the AE signal energy attenuation rate of soft wood was more sensitive to the change of frequency. This is because soft wood has a more uniform structure compared to hard wood [35], as shown in Figure 11.

To quantitatively characterize the degree of energy attenuation of AE signals, the actual distance of AE energy attenuation to 50% and 90% was used to characterize the effect of different frequencies of AE signals on energy attenuation, and the attenuation distances of AE signals at different frequencies could be calculated as shown in Table 6.

Table 6 shows the energy attenuation distances of AE signals for each frequency in different specimens. As can be seen from the table, the AE signal energy decreased rapidly to 50% at the beginning of the attenuation, but the distance increased significantly when the remaining energy attenuated to 10%. At the same frequency, the energy attenuation distance of hard wood was smaller than that of soft wood, with the farthest propagation distance of 192.94 mm for hard wood and 207.57 mm for soft wood. This was due to the fact that the AE signal propagated mainly along the fiber direction in wood, and, therefore, in hardwoods with shorter fibers [36], the AE signal energy attenuated more quickly.

## 4. Conclusions

The test used an arbitrary waveform generator to generate different frequency pulse string signals to simulate AE sources, studied the amplitude and energy attenuation law of AE signals propagating along the textured direction of *Ulmus pumila*, *Zelkova schneideriana*, *Cunninghamia lanceolata*, and *Pinus sylvestris var. mongolica*, and the effects of frequency on the amplitude and energy attenuation characteristics of AE signals in different specimens. The results obtained were as follows:

(1) Both the amplitude and energy of AE signal showed exponential attenuation with the increase of propagation distance, and the change of energy level of AE source had no significant effect on its amplitude and energy attenuation law at the same frequency.
(2) Both the amplitude and energy attenuation rate of AE signal increased with the increase of frequency, and the increase gradually slowed down. The change of AE signal energy attenuation rate of soft wood was greater than that of hard wood, which indicated that the energy attenuation of soft wood was more sensitive to change of frequency.
(3) In the process of AE signal energy attenuation, the distance used when the energy was attenuated to 50% was shorter, while the attenuation distance increased significantly when the remaining energy was attenuated from 50% to 10%. However, the attenuation distance of AE signal of the same frequency in hard wood was smaller than that in soft wood, which was because the AE signal propagated mainly along the fiber direction in wood, and the fiber of hard wood is generally smaller than that of soft wood.

This article studied the effect of frequency on the amplitude and energy attenuation of AE signal. The results show that the change of AE signal frequency has a significant impact on its attenuation characteristics, especially in the low frequency stage, and soft wood is more sensitive. An energy attenuation model of AE signals with different frequencies was established, which can be applied to distinguish wood types, and damage localization of wood.

In this paper, only the effect of frequency on the attenuation characteristics of the AE signal as it propagated along the wood in the direction of the grain was studied, and the subsequent study of the changes in the attenuation characteristics of the AE signal in different propagation directions of the wood could be continued. The AE signal could be decomposed to further study changes in the attenuation characteristics of body waves at different frequencies.

## Figures and Tables

**Table 1:** Test piece parameters.

Test Pieces	Material	Annual Rings	Moisture Content (%)	Wood Density (g/cm^3^)
T1	*Ulmus pumila*	50	11.6	0.62
T2	*Zelkova schneideriana*	100	11.4	0.61
T3	*Cunninghamia lanceolata*	10	11.1	0.39
T4	*Pinus sylvestris var. mongolica*	15	11.3	0.49

**Table 2:** Test apparatus.

Equipment	Model	Characteristics
Wood density tester	DA-900CE	Measurement range: 0.001~99.999 g/cm^3^ Dimension (mm): 4250 × 1750 × 3250
Dry box	101-0EBS	Temperature control range: RT + 10~250 °C Dimension (mm): 350 × 350 × 350
Acquisition card	NI USB-6366	Sample rate: 0~2 MHz 8 AI, 24 DIO, 2 AO
Sensors	RS-2A	Frequency range: 50~400 kHz Temperature: −20~130 °C
Signal amplifier	PA I	Gain: 40 dB Dimension (mm): 116 × 36 × 30
Arbitrary waveform generator	SDG805	Sample rate: 125 MSa/s Frequency Specification: 500 μHz ~ 5 MHz Output Specification: 4 mV~20 V

**Table 3:** Fitting parameters of AE signal energy attenuation of T1 specimen.

Frequency (kHz)	*K*	*b*	*α*	*β*
1	1.99	−2.52	0.0065	0.0126
5	2.05	−2.82	0.0045	0.0129
10	2.05	−2.59	0.0056	0.0129
30	2.05	−2.57	0.0058	0.0129
50	2.05	−2.59	0.0056	0.0130
150	2.07	−2.61	0.0054	0.0131

**Table 4:** Fitting parameters of AE signal energy attenuation of T3 specimen.

Frequency (kHz)	*K*	*b*	*α*	*β*
1	1.75	−2.21	0.0120	0.0111
10	1.85	−2.55	0.0075	0.0117
30	1.89	−2.39	0.0084	0.0120
50	1.90	−2.36	0.0085	0.0120
150	1.91	−2.37	0.0083	0.0121

**Table 5:** The increase rate of attenuation rate *η*.

Frequency (kHz)	T1	T2	T3	T4
10	2.58%	2.04%	5.26%	5.11%
30	2.75%	3.93%	7.84%	7.95%
50	3.40%	4.43%	8.14%	8.52%
150	3.79%	4.93%	9.02%	9.66%

**Table 6:** AE signal energy attenuation distance.

Frequency (kHz)	T1		T2		T3		T4	
	50% (mm)	90% (mm)	50% (mm)	90% (mm)	50% (mm)	90% (mm)	50% (mm)	90% (mm)
1	55.07	182.92	58.08	192.94	62.49	207.57	62.27	206.86
10	53.77	178.64	56.92	189.08	59.37	197.22	59.24	196.80
30	53.59	178.02	55.88	185.63	57.95	192.52	57.71	191.72
50	53.25	176.88	55.62	184.76	57.79	191.96	57.38	190.61
150	53.05	176.24	55.34	183.85	57.31	190.39	56.84	188.80

50%: energy attenuation to 50% propagation distance, 90%: energy attenuation to 90% propagation distance.

## Data Availability

Not applicable.
